# Case Report: When cystic fibrosis, elexacaftor/tezacaftor/ivacaftor therapy, and alpha1 antitrypsin deficiency get together

**DOI:** 10.3389/fped.2024.1378744

**Published:** 2024-04-09

**Authors:** Rachel Kinuani, Jessica Ezri, Yann Kernen, Isabelle Rochat, Sylvain Blanchon

**Affiliations:** ^1^ Pediatric Pulmonology and Cystic Fibrosis Unit, Department Women-Mother-Child, Service of Pediatrics, Lausanne University Hospital and University of Lausanne, Lausanne, Switzerland; ^2^ Department of Pediatrics, Divisions of Pediatrics Pulmonology, University Hospital Liège, Liège, Belgium; ^3^ Pediatric Gastro-Enterology Unit, Department Women-Mother-Child, Service of Pediatrics, Lausanne University Hospital and University of Lausanne, Lausanne, Switzerland; ^4^ General Pediatric Private Practice, Yverdon-les-Bains, Switzerland

**Keywords:** cystic fibrosis, CFTR modulator, ELX/TEZ/IVA, CF liver disease, drug-induced liver injury, Z allele, *SERPINA1*, children

## Abstract

In the last 10 years, the care of patients with cystic fibrosis (CF) has been revolutionized with the introduction of *cystic fibrosis transmembrane conductance regulator* (CFTR) modulator drugs, with a major impact on symptoms and life expectancy, especially considering the newest and highly effective elexacaftor/tezacaftor/ivacaftor (ELX/TEZ/IVA) therapy. Conversely, adverse effects are relatively frequent, with some being life-threatening, such as severe hepatitis. Clinical trials on children starting CFTR modulators have reported transaminase elevations >3× upper limit of the norm in 10%–20% of patients, whereas real-life studies have reported discontinuation rates three times higher than those observed in phase 3 trials. We report the case of a 10-year-old boy with CF who developed severe acute hepatitis 2 weeks after starting ELX/TEZ/IVA therapy. An extensive screening for potential causes led to the identification of heterozygous alpha1-antitrypsin (AAT) deficiency with genotype MZ. The Z allele of *SERPINA1* gene, encoding AAT, is known as a risk factor for CF liver disease. We hypothesized that it may act as a risk factor for drug-induced liver injury from CFTR modulators, notably ELX/TEZ/IVA. Therefore, checking AAT before starting CFTR modulator therapy can be suggested, in particular for children with previous, even transient, liver disease.

## Introduction

Cystic fibrosis (CF) is caused by biallelic variants in *cystic fibrosis transmembrane conductance regulator* (CFTR) gene encoding for the CFTR protein. Affecting 100,000 patients worldwide, CF is a systemic disease, notably involving the lungs, the digestive tract, and the liver (1). In 10%–40% of the patients with CF (pwCF), CF liver disease (CFLD) is considered when at least two of the following criteria are met: (1) abnormal physical examination, (2) transaminase and gamma-glutamyl transferase (GGT) levels above the upper limit of the norm (ULN) on at least three consecutive occasions over 12 months, after exclusion of other causes, and (3) ultrasonographic (US) evidence of liver involvement (2). Finally, a liver biopsy can be performed to confirm CFLD. A large French observational study reported the incidence of CFLD to be continuously increasing throughout the lifetime, with a yearly rise of about 1% from birth, reaching 32% by the age of 25 and stabilizing thereafter. There is a statistical association with male sex, homozygous F508del genotype, and a history of meconium ileus (3). In addition, an international study on genetic modifiers has identified the Z allele of *SERPINA1* gene, associated with alpha-1 antitrypsin (AAT) deficiency, as a risk factor for CFLD (4).

Over the last 30 years, the life expectancy of pwCF has slowly increased to 45–50 years, owing to improvements in symptomatic treatment and patient care (5). In the last 10 years, “CFTR modulators” have led to a paradigm shift in CF care by directly targeting the dysfunctional protein. These include “CFTR potentiators” such as ivacaftor (IVA), which increase the ability to keep the chloride channel open, used alone or in addition to “CFTR correctors,” such as lumacaftor (LUM), tezacaftor (TEZ), and elexacaftor (ELX), which facilitate processing, trafficking, and stabilization of the protein at the cell membrane. To date, four drugs are available, notably LUM/IVA for patients with homozygous F508del variant and ELX/TEZ/IVA for patients with at least one F508del variant. Therefore, CFTR modulators are revolutionizing CF care with a major impact on symptoms and life expectancy, especially considering the latest and highly effective ELX/TEZ/IVA drug (6, 7). On the other hand, the adverse effects (AEs) of CFTR modulators are relatively frequent, as evidenced in a real-world safety review (6) and in a more recent systematic review focused on ELX/TEZ/IVA in patients aged 6 years or older (7).

We report the case of a child with CF who developed severe acute hepatitis after starting ELX/TEZ/IVA therapy, which led to the identification of heterozygous AAT deficiency.

## Case report

**Figure 1:**
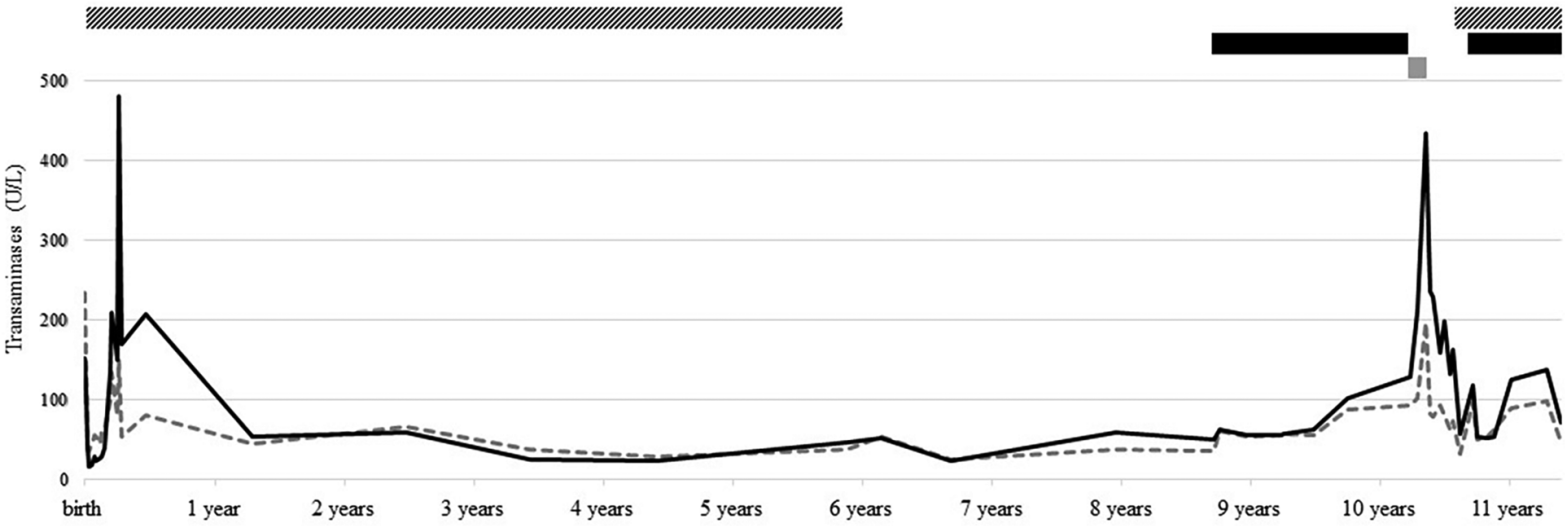
Follow-up of transaminase [i.e., aspartate aminotransferase (AST, dotted gray line) and alanine aminotransferase (ALT, solid black line)] levels, from birth to present, and treatment with UDCA (striped bar), LUM/IVA (black bar), and ELX/TEZ/IVA (gray bar).

This 10-year-old boy was full-term born after the discovery of gastroschisis at 22 weeks gestation. At birth, he presented with large gastroschisis involving herniation of the left liver, small bowel, and colon, requiring staged reduction by silo, followed by patch closure, before final closure when he was 2 months old. At 1 month of age, enteral feeding was started, but the child developed intestinal obstruction due to terminal ileum impaction compatible with meconium ileus, leading to ileostomy finally closed at 5 months of age. In the meantime, newborn screening was positive for CF, and genetic testing revealed a homozygous F508del *CFTR* variant. CF treatment was started according to standards of care. By day 20, the child developed persisting cholestasis (Figure 1), attributed to the gastroschisis surgery, CF, and prolonged parenteral nutrition. On day 25, ursodeoxycholic acid (UDCA) 25 mg/kg/day was started, with a very slow decrease in cholestasis parameters.

After that, he had regular follow-ups in our CF clinic without any relevant events. At 5.5 years old, after more than 5 years of maintaining normal liver parameters, UDCA was discontinued. At 8.5 years old, while liver function tests were still normal, CFTR modulator LUM/IVA was started, allowing clinical and lung function improvement. LUM/IVA was well tolerated despite an isolated two- to threefold increase in transaminases without cholestasis or US anomaly. At 10 years old, the boy transitioned to ELX/TEZ/IVA following its approval in Switzerland. From day 10, transaminases progressively increased to reach 10× ULN after 4 weeks, accompanied by concomitant nausea and fatigue. ELX/TEZ/IVA was discontinued, and symptoms quickly subsided, but transaminases remained three- to fourfold increased 2.5 months after discontinuation. Causes of liver cytolysis, such as infections, metabolic disorders, and endocrinological diseases, were exhaustively excluded. Due to slightly decreased plasma AAT (0.86 g/L, N > 0.9), genetics analysis revealed the presence of alleles M and Z of *SERPINA1* gene. UDCA at a dosage of 15 mg/kg/day was introduced, resulting in rapid nearly normalization (1–2× ULN) of hepatic parameters. After a discussion with the family, who were reluctant to resume ELX/TEZ/IVA with a decreased dosage, the child reverted to LUM/IVA. After 1 year, he remains asymptomatic, with transaminase levels stabilized at 2–3× ULN and no evidence of cholestasis or US abnormalities.

## Discussion

AAT deficiency, caused by autosomal recessive mutations in *SERPINA1* gene encoding for AAT, is recognized as a risk factor for CFLD (4, 8). We hypothesized that it may also act as a risk factor for drug-induced liver injury (DILI) during CFTR modulator treatment, notably ELX/TEZ/IVA. AAT, an abundant serum protein synthesized in the liver, acts as the primary inhibitor of neutrophil elastase. More than 400 variants of *SERPINA1* gene are described, including the most common wild type “M” and the most common mutants “*S*” and “*Z*.” The *Z* variant is overwhelmingly associated with liver disease, given the protein's inability to fold into its final conformation, leading to accumulation in liver cells, intracellular damage, and finally chronic liver disease (3). The Z allele is reported as a risk factor for CFLD (4, 8), strongly associated with severe hepatopathy with portal hypertension (odds ratio: 5) (4), with a cumulative incidence of CFLD reaching 47% by the age of 25 compared with 30% in non-carriers (3). Furthermore, Jaspers et al. reported the only known case of a patient with both CF and total AAT deficiency (homozygous ZZ), leading to persistent and severe cholestasis necessitating liver transplantation at the age of 8 months (9). Conversely, no significant effect was found for the S allele (3). Indeed, a poor concordance of liver disease in sibling pwCF and the heterogeneous phenotype of CFLD argue against a major role of environmental factors and suggest that non-CFTR genetic variations contribute to the heterogeneity of CFLD, thus called “modifier genes.” To date, *SERPINA1* is the only modifier gene identified by a large two-stage study (8). However, the low prevalence of the Z allele in pwCF compared with the high prevalence of CFLD suggests that other modifier genes may exist (8, 10).

To our knowledge, no publication has yet given insights into AAT deficiency as a potential risk factor for DILI, even more so in pwCF. DILI is an acute or chronic liver injury, manifesting as hepatitis, cholestasis, or a combination of both, secondary to drugs or herbal compounds. DILI is classified as either predictable, stemming from the direct toxicity of the drug and classically dose-related with few days of latency, or idiosyncratic. Most of the DILI cases are idiosyncratic, meaning they are unpredictable/unexpected, with unclear mechanisms that may include immune- or non-immune-mediated mitochondrial injuries in hepatocytes, depending on host factors (age, sex, genetic polymorphism notably of CYP450 enzymes, etc.), drugs (lipophilicity, dosage, etc.), and environmental factors (alcohol, tobacco, hepatotoxic substances, etc.) (11–14). Population-based studies reported the incidence of DILI ranging from 13.9 (France, 1997–2000) to 19.1 (Iceland, 2010–2011) cases per 100,000 inhabitants (15, 16); however, its incidence in pwCF is unknown, although it has been reported as a frequent AE associated with CFTR modulators (6, 7).

For LUM/IVA, a phase 3 clinical trial comparing LUM/IVA to placebo in patients aged 12 years or older showed a similar incidence of AEs and transaminase elevations >3xULN in both groups; serious AEs related to DILI were only reported in the LUM/IVA group (0.9%). After discontinuing LUM/IVA, all patients exhibited normalization of liver function (17). A phase 3 clinical trial involving children aged 6–11 years also revealed a similar incidence of AEs in both groups, but there was a more frequent increase in transaminase levels in the LUM/IVA group (13% vs. 8%) (18). An extension study (up to 96 weeks) involving children aged 6–11 years reported 18% of children with transaminase levels >3× ULN and 4% of children discontinuing the treatment due to AEs (elevated transaminase levels, urticaria, gastrointestinal events, etc.) (19). Importantly, a review of real-life studies reported a discontinuation rate three times higher than that observed in the phase 3 trials (18.2% vs. 5%), mainly related to respiratory AEs (20).

For ELX/TEZ/IVA, in the phase 3 clinical trial involving patients aged 12 years or older, AEs were comparable in ELX/TEZ/IVA and TEZ/IVA groups, without reported discontinuations. Transaminase levels >3× ULN were only observed in the ELX/TEZ/IVA group (7%) (21). Recently, a meta-analysis reported transaminase levels >3× ULN in 6%–10% of patients and bilirubin >2× ULN in 0%–3% of patients (7). In children aged 6–11 years, DILI seemed slightly more prevalent, with transaminase levels >3× ULN in 10.6%–13.6% of patients and >5× ULN in 1.5%–5.1% (vs. 4.9% and 1.6% in the placebo group, respectively); however, no discontinuations related to DILI were reported (22). Finally, despite being frequent, most AEs are transient and do not require discontinuation of the drug. Nevertheless, case reports of severe DILI cases have been published, such as this 21-year-old patient who developed increased transaminases, hyperbilirubinemia, US-detected liver abnormalities, and characteristic lesions on liver biopsy 5 months after starting ELX/TEZ/IVA. Discontinuation of the drug led to the normalization of blood tests after 6 months (23).

Considering the high frequency of DILI associated with CFTR modulators in adults and children, specific prescribing information from manufacturers and recommendations from national drug agencies have been published. Therefore, transaminase levels are systematically measured before introducing any CFTR modulator therapy and rechecked at least every 3 months during the first year of treatment. Furthermore, if transaminase levels reach 5xULN or 3xULN and bilirubin levels reach 2× ULN, treatment should be discontinued, at least temporarily. If elevated transaminase levels are resolved, and after assessing benefits vs. risks, the possibility of resuming modulator treatment at either the full or reduced dosage should be discussed.

Our patient developed mild DILI under LUM/IVA, followed by severe DILI under ELX/TEZ/IVA, revealing heterozygous MZ variants for AAT. Retrospectively, hepatic cytolysis during the initial months of life was potentially explained by this genotype, known to cause liver injury, including CFLD, and worsen liver injury from other causes. However, the question remains open as to why our patient experienced severe hepatic cytolysis under ELX/TEZ/IVA, while transaminase levels only slightly increased under LUM/IVA. A retrospective study found that children with CFLD treated with LUM/IVA experienced significant decreases in GGT, transaminase-to-platelet index, and GGT-to-platelet ratio, which is correlated with imaging-based markers of liver fibrosis; in contrast, no improvement was observed in children treated with ELX/TEZ/IVA (24). This beneficial effect of LUM/IVA on CFLD has been observed in a previous study (25). There is no explanation at this point, while ELX/TEZ/IVA does not have a similar beneficial effect on CFLD. The undergoing PROMISE study might provide insight into it.

## Conclusion

In light of this first case report highlighting partial AAT deficiency in the context of DILI associated with ELX/TEZ/IVA, screening for DILI risk factors (and notably AAT deficiency) before the introduction of recently marketed ELX/TEZ/IVA can be suggested, in particular for patients with a previous history of even transient CFLD. Nevertheless, our hypothesis should be confirmed/statistically validated in a larger, optimally multicentric, cohort*.* Our next step will involve screening for AAT deficiency in all patients with hepatic anomalies after starting CFTR modulators to strengthen the hypothesis regarding the association between AAT deficiency and DILI risk.

## Data Availability

The original contributions presented in the study are included in the article/Supplementary Material, further inquiries can be directed to the corresponding author.
